# Portuguese validation of the Self-Generated Stress Scale

**DOI:** 10.1192/j.eurpsy.2022.1705

**Published:** 2022-09-01

**Authors:** M.J. Brito, F. Carvalho, P. Vitória, A.P. Amaral, M. Carneiro, C. Cabacos, A. Araújo, A. Macedo, A.T. Pereira

**Affiliations:** 1 Centro Hospitalar e Universitário de Coimbra, Department Of Psychiatry, Coimbra, Portugal; 2 Faculty of Medicine of University of Coimbra, Institute Of Psychological Medicine, Coimbra, Portugal; 3 University of Beira Interior, Department Of Psychology And Education, Covilhã, Portugal; 4 ISCTE - University Institute of Lisbon, Cis-iul - Centre For Social Research And Intervention, Lisboa, Portugal; 5 Polytechnical Institute of Coimbra, Coimbra Health School, Coimbra, Portugal; 6 Coimbra Institute for Biomedical Imaging and Translational Research, -, Coimbra, Portugal

**Keywords:** self-generated stress, personality, psychological distress, emotional regulation

## Abstract

**Introduction:**

Self-Generated Stress might be defined as stress that is created by oneself by engaging in behavior or making decisions that ultimately add strain to pre-existing personal stress. The Self-Generated Stress Scale (SGSS; Flett et al. 2020) is a seven-item self-report measure built to assess this tendency to make one’s own life more stressful.

**Objectives:**

To analyze the psychometric properties of the Portuguese Version of the SGSS.

**Methods:**

Participants (127 medicine and dentistry students; 78.0% female) answered an online survey including the preliminary Portuguese version of the SGSS and other validated questionnaires: Maslach Burnout Inventory – Students Survey, Depression Anxiety and Stress Scales, HEXACO-60 and *Big Three Perfectionism Scale.*

**Results:**

Confirmatory Factor Analysis showed that the unidimensional model presented good fit indexes (χ^2^/df=1.546; RMSEA=.0666, p<.001; CFI=.982 TLI=.972, GFI=.960). The Cronbach’s alfa was .868. Pearson correlations between SGSS and the other measures were significant (p<.01) and moderate/high: Burnout, .412; Stress/Anxiety/Depression, >.550; Perfectionism, .600; Emotionality, .315; Extroversion, -.411. After controlling for the effect of Emotionality and Extroversion, SGSS explained significant additional increments of 19.9% and 14.0% of the DASS and MBI variance; controlling for Perfectionism, the increments were respectively of 27.9% and 2.0%. SGSS mean score (22.96±5.90 was not significantly different by gender.

**Conclusions:**

As observed with the original English-language scale, the Portuguese version of SGSS showed good validity (construct and convergent-divergent) and internal consistency. As such, the SGSS might be useful in further investigation, particularly to explore the different pathways between personality traits, emotional regulation processes and psychological distress.

**Disclosure:**

No significant relationships.

